# Outpatient follow-up of tumour diseases through video-based value-oriented behavioural activation (ViVA): study protocol for a randomised controlled trial

**DOI:** 10.1186/s13063-024-07953-w

**Published:** 2024-02-14

**Authors:** Maren Reder, Christine Hofheinz, Lena Melzner, Gabriele Prinz, Christoph Kröger

**Affiliations:** Institute of Psychology, University of Hildesheim, Universitätsplatz 1, Hildesheim, 31141 Germany

**Keywords:** Value-oriented behavioural activation, Psycho-oncological aftercare, Oncological patients, Psychological distress, Covariate-adaptive randomisation, Video-based treatment

## Abstract

**Background:**

In Germany, approximately half a million people are diagnosed with cancer annually; this can be traumatic and lead to depression, anxiety, and adjustment disorders necessitating psycho-oncological intervention. Value-oriented behavioural activation, adopted from depression psychotherapy, aims to provide structured support to help patients adjust their personal values, goals, and activities within the context of their changed life situation. This trial aims to evaluate the effectiveness of video-based value-oriented behavioural activation against German S3-Guideline-compliant aftercare for cancer patients dealing with psychological distress.

**Methods:**

This trial will use covariate-adaptive randomisation according to gender and type of tumour disease to assign participants to one of two study arms (value-oriented behavioural activation consisting of 12 manualised follow-up sessions delivered via video consultation vs. S3-Guideline-compliant aftercare comprising three supportive talks). Psychological strain, psychosocial distress, quality of life, work-related outcomes, fear of cancer recurrence, goal adjustment, satisfaction with the consultant-participant relationship, and rumination will be measured at baseline, twice during treatment, posttreatment, and at the 6-month follow-up. The target sample of 146 tumour patients experiencing high psychosocial distress will be recruited at the Rehazentrum Oberharz, Germany.

**Discussion:**

This trial aims to test the effectiveness of value-oriented behavioural activation in aftercare for tumour patients, focusing on its capacity to reduce distress and the potential for long-term effects evaluated through a 6-month follow-up. The study’s possible challenges include enrolling a sufficient sample and ensuring adherence to treatment, mitigated through in-person recruitment and rigorous training of staff. If successful, the results will be of high public health relevance, especially for psychotherapeutic care in rural areas and among patients with limited mobility considering the video-based approach of the trial.

**Trial registration:**

This study was registered at the German Clinical Trials Register: DRKS00031900 on Sep 19, 2023.

**Supplementary Information:**

The online version contains supplementary material available at 10.1186/s13063-024-07953-w.

## Background

In Germany, every year approximately half a million people are newly diagnosed with cancer [1]. A diagnosis of a tumour disease can be regarded as a potentially traumatic event [2]. In some cases, a psychological impairment develops that meets the criteria for a mental disorder. Prevalence estimates for these resulting mental disorders, such as depression (up to 58 %), anxiety disorders (up to 49 %), and adjustment disorders (up to 52 %) vary depending on the type of tumour disease and the associated functional limitations and pain as well as the stage of the disease [3, 4]. Therefore, according to the current German S3-Guideline [5], once an increased psychological burden has been identified, a psycho-oncological intervention should be offered.

Currently, very unspecific, nonsystematic psycho-oncological interventions are offered in Germany [6]. These interventions have achieved moderate short- and medium-term effects on self-reported anxiety, depression, and quality of life [7]. Unspecific techniques (e.g. psycho-educational information and relaxation techniques) do not guarantee individual adaptation to the needs and requirements of persons with tumour disease. Especially when facing a potentially life-threatening disease, highly individualised needs will be involved, and those affected will have to adapt their goals, especially with a shortened life expectancy. Individualised goal-value-clarification and adaptation of treatment to these can be found in various treatment manuals for affective disorders [8], but these interventions have not yet been employed for psycho-oncological treatment [7]. It has been shown that successful detachment from goals that have become unattainable and a stronger focus on attainable goals are positively associated with the well-being of patients with tumours [9]. Manualised value-oriented behavioural activation may be a solution and result in better short- and long-term effects regarding anxiety, depression, and quality of life.

According to a meta-analysis [10], various approaches to value-oriented behavioural activation (VA) for individuals with depressive disorders proved to be at least equivalent to the conventional approach of cognitive-behavioural treatment of depression. A randomised controlled trial showed that VA also leads to a clinically significant reduction in depressive symptoms and perceived physical limitations and an improvement in quality of life and level of functioning in patients with breast cancer and an acute depressive episode [11]. Furthermore, this form of VA can promote the restoration of a sense of control in patients through its explicit reference to personal values and the resulting behavioural reinforcement [12]. Although previous research on VA in tumour diseases has been conducted predominantly with female patients with breast cancer, initial findings indicate that VA is an effective intervention for other oncological target groups [13].

VA is established for the treatment of depression [10] by clarifying personally important values and life goals against the background of a changed life situation and in (re)orienting everyday life accordingly. A detailed description of VA is available in a German-language manual for depressive disorders [14]. Applying a value-oriented approach borrowed from depression treatment to psycho-oncological care enables highly individualised treatment.

Current psycho-oncological interventions require participants to be motivated, mobile, and, above all, physically able to see consultants on an outpatient basis. This cannot be considered true for all participants, especially since oncological treatment is often accompanied by a number of persistent side effects such as pain, fatigue, and sleep disorders [15]. By using a video consultation, it can be ensured that participants and consultants communicate synchronously and that participants receive support in coping with everyday life despite these challenges.

The present study aims to assess the effectiveness of video-based, value-oriented behavioural activation (in the VA-V group) in comparison to German S3-Guideline-compliant outpatient aftercare (in the guideline group) in a randomised controlled trial. The primary objective is to evaluate whether the VA-V results in lower levels of distress (i.e. depression and anxiety scores on the HADS). The secondary objectives are to evaluate whether the VA-V (1) improves ability level and quality of life, (2) improves goal adjustment, (3) yields sufficient levels of treatment satisfaction and working relationships in a process evaluation, and (4) is affected by background factors (e.g. disease prognosis) in its influence on primary and secondary outcomes.

## Methods/design

### Trial design

**Fig. 1:**
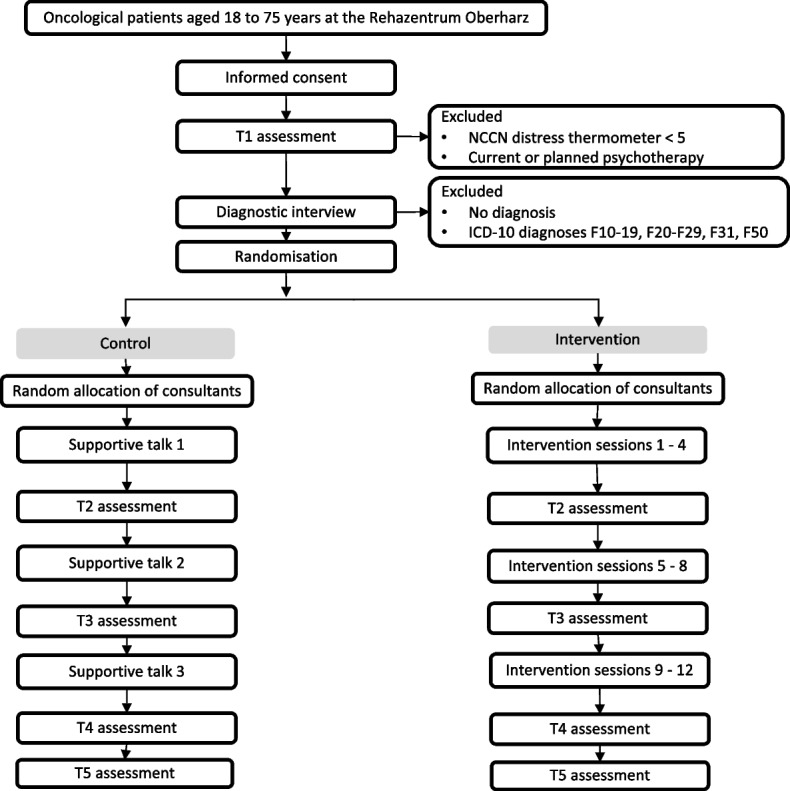
Trial flow diagram

This study is a nonblinded two-armed parallel randomised controlled trial (see Additional File 1 for the SPIRIT checklist). The superiority of the VA-V over the German S3-Guideline-based intervention will be evaluated. Participants will be randomly assigned to either the VA-V group or the German S3-Guideline-compliant aftercare group. Random allocation will be performed on a 1:1 ratio basis and covariate-adaptively for gender and type of tumour disease. The assessments will be conducted at baseline (T1), during the intervention (T2, T3), postintervention (T4), and at the 6-month follow-up (T5; see Fig. 1). The VA-V group will receive 12 online intervention sessions. The control group will be an active control group receiving 3 supportive online talks according to the German S3-Guideline [5].

### Participants, interventions, and outcomes

#### Study setting

Participants will be recruited at the Rehazentrum Oberharz Clausthal-Zellerfeld, Lower-Saxony, Germany, a clinic specialising in the rehabilitation of cancer patients (with the exception of lung cancer, cancers of the head and neck area, brain tumours and cancers of the nervous system). The sponsor of Rehazentrum Oberharz is the Deutsche Rentenversicherung (German pension insurance).

#### Eligibility criteria

The inclusion criteria will be the presence of an oncological disease (irrespective of type or prognosis; C00-D48), experiencing high psychosocial distress (defined as $\ge 5$ on the National Comprehensive Cancer Network (NCCN) Distress Thermometer [16]), and having a mental disorder according to the International Statistical Classification of Diseases and Related Health Problems (ICD-10 [17]). Correspondingly, individuals with the following diagnoses according to the ICD-10 will be included: unipolar depressive disorders (F32 – F34), anxiety disorders (F40, F41), stress-related and somatoform disorders (F42, F43, F45, F48.0), obsessive-compulsive disorders or adjustment disorders (F43.2), and nonorganic sleep disorders (F51). The minimum age will be 18 years, the maximum age will be 75 years. Participants will need to have sufficient knowledge of the German language to participate in the study, be able to give consent (e.g. persons under guardianship cannot participate), and have provided written informed consent (incl. consent to videography for quality assurance).

The exclusion criteria will be bipolar disorders (F31), schizophrenia and other delusional disorders (F20-F29), substance disorders (F10-19), and eating disorders (F50) because these disorders cannot be regarded as a consequence of the tumour disease in a narrow sense and require disorder- and symptom-specific therapeutic techniques which cannot be provided within the manualised VA-V intervention. Current or planned psychotherapeutic treatment will also be an exclusion criterion because this could represent a confounder in the evaluation of the intervention effect.

#### Interventions

*VA-V intervention.* Participants in the VA-V will receive twelve manual-based 50-minute sessions of video-based value-oriented behavioural activation, usually once a week, offered by a trained consultant. The trained consultants will be randomly assigned to the participants. However, due to holidays, illness, or other appointments, there may be 2–3 weeks between two appointments. Both the VA-V and the German S3-Guideline-based intervention will be offered by the same consultants to minimise person effects. If an appointment has been missed, efforts will be made to contact the participant to schedule a new appointment. If a participant wishes to discontinue the intervention, participants will be asked for their reasons. Discontinuations and all deviations of the intervention protocol will be documented.

To ensure treatment integrity and adherence to the intervention protocol, the intervention will be carried out by specially trained scientific staff (psychologists and pedagogues) of the Department of Clinical Psychology and Psychotherapy based on the VA-V manual and supervised by a psychological psychotherapist. Training and supervision will be provided by the author of the VA-V manual (CH), a psychological psychotherapist. In addition, it is planned that the conducting scientific staff will participate in a specific curriculum for psycho-oncological qualification―the advanced training in psychosocial oncology (Weiterbildung Psychosoziale Onkologie) certified by the German Cancer Society―during the project period. In the VA-V condition, all sessions will be video-graphed. The videographies will be used for quality assurance in terms of treatment integrity and supervision and―if the participants separately consented―for anonymised analysis of specific session parts (e.g. a thought experiment).

Video sessions will be implemented through the video conferencing tool RED connect (RED Medical Systems GmbH). This is a secure, end-to-end encrypted communication platform specifically designed and approved for the medical-therapeutic field. Data exchange (encrypted several times) is realised exclusively between consultants and participants (peer-to-peer connection). In the following, the 12 intervention sessions are described.

1. *Introduction and anamnesis.* In the first session, the consultant and the study will be introduced, and relationship building, clarifying the organisational framework, and anamnesis of life and medical history will be conducted.
2. *Feedback on diagnostic assessments and psychoeducation.* The most important results of the diagnostic assessments will be discussed with the participants. Myths about cancer as well as associated hopes (e.g. the feeling of being able to gain control if cancer can be influenced by positive thinking) and costs (e.g. the feeling of being to blame for one’s own illness) will be disputed. Furthermore, the extent to which current psychological distress is a consequence of the disease and the treatment or its side effects will be explored via psychoeducation.
3. *Value-goal clarification and introduction to values and the value questionnaire.* The concept of values in life (according to Acceptance and Commitment Therapy [18]) will be introduced. This will be followed by an exploration of the participants’ own values before the cancer diagnosis and after the diagnosis and treatment (importance rating from 1 to 10); changes in values will be discussed.
4. *Elaboration of old and new values, goal 1.* In this session, the most important life values will be selected by the participants. This will be followed by elaboration of the previous status or situation before the diagnosis and assessment of goals and activities based on the current life situation according to a three-colour scheme (green for current and possible goals/activities, red for goals/activities that are irretrievably blocked or no longer important, yellow for goals/activities that are no longer possible in the previous implementation or whose priority has decreased in comparison to before). New goals and activities will be developed, and value-based activities will be planned.
5. *Elaboration of old and new values, goal 2.* Like in the last session, more important value areas will be elaborated, and value-based activities will be planned.
6. *Elaboration of old and new values, goal 3, thought experiment.* Like in the last two sessions, more important value areas will be elaborated, and value-based activities will be planned. Additionally, a thought experiment will be conducted. Participants will be asked to imagine that they only have 6 months to live. It will then be elaborated how they would like to use their time with regard to the selected areas of life, values, goals, and activities (i.e. would there be differences/changes in priorities?).
7. *Value-based behavioural activation, further activity building, and dealing with problems.* Barriers to the implementation of activities will be collected. Value-based activities will be planned (with special attention given to the activating amount of activities).
8. *Completing the individual activity plan.* Exhaustion or fatigue syndrome, as a special barrier to the implementation of activities, will be discussed. From this, concrete changes for everyday life will be derived. Additionally, dealing with the avoidance of anticipated consequences will be a focus topic. The short- and long-term consequences of avoidance behaviour will be determined. Subsequently, corresponding activities that have been avoided thus far will be planned.
9. *Dealing with specific problems, problem-solving training 1.* Problem-solving training will be introduced. First, it will involve a problem and goal definition. Second, possible solutions will be developed regardless of how unusual they are. Third, these possible solutions will be evaluated. Fourth, the best possible solution will be chosen. The fifth step will be to plan the concrete implementation of this possible solution.
10. *Problem-solving training 2.* In the sixth step of the problem-solving training, solution attempts since the last session will be reviewed and evaluated. Sessions 10 and 11 will be designed according to the individual problems/symptoms of the participant. Either further problem-solving training, dealing with rumination, or dealing with fear of cancer recurrence will be the focus topics of these sessions. Dealing with rumination will include defining rumination and identifying it as an unfavourable coping strategy. Positive and negative meta-thoughts, dealing with trigger thoughts and rumination suspension will be disputed. Dealing with the fear of cancer recurrence will include defining fear of cancer recurrence and identifying positive meta-thoughts, which will then be disputed. The acceptance of unpleasant feelings and the reduction of safety/avoidance behaviour will be used as strategies.
11. *Problem-solving training 3.* The session will be conducted according to the problem focus of the participant and can include either further problem-solving training or dealing with rumination or fear of cancer recurrence.
12. *Closing session, summary, relapse prevention.* The various main topics of the intervention (clarification of values and goals, development of value-oriented activities, problem-solving, dealing with rumination and/or fear of cancer recurrence if necessary) will be summarised and reflected upon. An emergency plan will be developed.

*German S3-Guideline-based intervention.* Participants in the active control group will receive aftercare according to the German S3-Guideline ‘Psycho-oncological diagnosis, counselling and treatment of adult cancer patients’ [5]. They will receive three supportive talks at roughly the same times as the intervention group will receive sessions 1, 6, and 12. Supportive talks will cover fatigue management and relaxation techniques.

The German S3-Guideline-based intervention will thus involve more comprehensive aftercare than care as usual (CAU), as large gaps in care exist in outpatient cancer counselling and in the provision of outpatient psycho-oncological and psychosocial services [19]. Many of the spatial planning regions in Germany are underserved or have a low coverage rate (54 % have a coverage rate < 50 %, 30 % have a coverage rate between 50 % and < 75 %) regarding outpatient psycho-oncological care [6].

#### Outcomes

Various empirically validated questionnaires will be used to collect the data. The participants will complete these questionnaires online on a survey portal (ViVA platform) specially designed for the study. The programming of the ViVA platform is based on LimeSurvey (https://www.limesurvey.org). The questionnaires themselves will be created in LimeSurvey, while the ViVA platform will enable a customised user interface for participants and research staff. For an overview of the study outcome measures, see Tables 1 and 2. Participants will be contacted via the channel they have chosen in advance (e-mail, SMS, or both).

**Table 1:** Measurement points and intervention sessions

Measurement/intervention	Content
Baseline measurement (T1)	Po-Bado-KF, NCCN Distress Thermometer, HADS, FBK-R23, SF-12, EQ-5D-5L, ERA^b^, WAI-r^b^, GoalAdjust, FCR7, RRQ
Intervention session 1^a^	Introduction and anamnesis
Intervention session 2	Feedback on diagnostic assessments and psychoeducation
Intervention session 3	Value-goal clarification, introduction to values and the value questionnaire
Intervention session 4	Elaboration of old and new values, goal 1
Intermediate measurement 1 (T2)	GoalAdjust, RRQ, ZUF-8 (only VA-V), WAI-SR (only VA-V)
Intervention session 5	Elaboration of old and new values, goal 2
Intervention session 6^a^	Elaboration of old and new values, goal 3
Intervention session 7	Value-oriented activation, further activity building and dealing with problems
Intervention session 8	Completing the individual activity plan
Intermediate measurement 2 (T3)	GoalAdjust, RRQ, ZUF-8 (only VA-V), WAI-SR (only VA-V)
Intervention session 9	Dealing with specific problems, Problem-solving training 1
Intervention session 10	Problem-solving training 2
Intervention session 11	Problem-solving training 3
Intervention session 12^a^	Closing session, summary, relapse prevention
Post measurement (T4)	HADS, FBK-R23, SF-12, EQ-5D-5L ERA^b^, GoalAdjust, FCR7, ZUF-8 (only VA-V), WAI-SR (only VA-V), RRQ, Closing questionnaire
Catamnesis (6 months post intervention, T5)	HADS, FBK-R23, SF-12, EQ-5D-5L, ERA^b^, GoalAdjust, FCR7, WAI-r^b^, RRQ

^a^ At roughly similar time points the 3 supportive talks in the German S3-Guideline group are scheduled. Content and duration deviate from the intervention group

^b^ Only participants who are not retired due to ill health or old-age

**Table 2:** Outcome measures

Measures	T1	T2	T3	T4	T5
Po-Bado-KF	x				
NCCN Distress Thermometer	x				
HADS	x			x	x
SF-12	x			x	x
EQ-5D-5L	x			x	x
FBK-R23	x			x	x
WAI-r^a^	x				x
ERA^a^	x			x	x
FCR7	x			x	x
GoalAdjust	x	x	x	x	x
WAI-SR (only VA-V)		x	x	x	
ZUF-8 (only VA-V)		x	x	x	
RRQ	x	x	x	x	x
Closing questionnaire				x	

The measures are listed in the order in which they are presented to the participants

^a^ Only participants who are not retired due to ill health or old-age

*Primary outcome:*

*Psychological strain.* Symptoms of general anxiety and depression will be assessed with the Hospital Anxiety and Depression Scale, German version (HADS-D) [20]. The 14 items (7 each for depresson and anxiety) will be rated on a four-point Likert scale.

*Secondary outcomes:*

*Psychosocial distress.* For the initial assessment, the German version of the NCCN Distress Thermometer [21] will be used as a measure of psychosocial distress. If participants meet the criterion on the NCCN Distress Thermometer, they will complete the Questionnaire on Stress in Cancer Patients (FBK-R23) [22] as a more specific measure. Twenty-three items indicate whether the problem currently applies (0 = *does not apply*) and to what extent (1 = *hardly at all* to 5 = *very strongly*). The items can be combined into one total score and five subscales (psychosomatic complaints, anxiety, information deficits, everyday restrictions, and social stress).

*Quality of life*. Health-related quality of life will be assessed using the short version of the SF-36 Health Survey, the Short Form 12 (SF-12) [23] and the 5-level EQ-5D version (EQ-5D-5L) [24]. The SF-12 comprises 12 items distributed across eight dimensions: general health perception (1 item), physical health (2 items), limited physical role function (2 items), physical pain (1 item), vitality (1 item), mental health (2 items), limited emotional role function (2 items), and social functioning (1 item). The items have different scale levels. A physical and a mental sum score can be calculated. The EQ-5D-5L covers mobility, self-care, usual activities, pain/discomfort, and anxiety/depression [24]. Each of these five dimensions has five levels of perceived problems (no problem, slight problems, moderate problems, severe problems, unable to/extreme problems). An index value will be derived from these profiles, allowing the calculation of quality-adjusted life years. Additionally, overall current health will be assessed on a vertical visual analogue scale (‘The best health you can imagine’ to ‘The worst health you can imagine’) [24].

*Work-related outcomes*. Using a short version of the Work Ability Index (WAI-r) [25], the ability to work will be assessed, and a prognosis can be made about the development of the ability to work in two years. For this purpose, the minimum standard of two items for recording absenteeism and work ability will be used. For self-efficacy expectations in relation to work, a German version of the Return-to-Work Self-efficacy Questionnaire [26]―the Expectations of Work (ERA) questionnaire [27]―will be used. It comprises eleven items (e.g. ‘I will be able to concentrate on my work’) with a six-point Likert scale ranging from 1 $=$ *totally disagree* to 6 $=$ *totally agree*. Work-related outcomes will be assessed only if participants are not retired (due to ill health or old-age).

*Fear of cancer recurrence*. A German version of the Fear of Cancer Recurrence 7 Scale (FCR7) [28] will be used to obtain information about the participants’ fear of disease recurrence. Participants will complete self-report measures of the FCR7 on the ViVA platform and the fear of cancer recurrence platform. This platform will serve as a follow-up measurement in which the participants will be asked daily about their fear of cancer recurrence (FCR) for a period of 10 days. The two data collection platforms (ViVA and FCR) will be independent of each other. The platform will technically and in terms of data protection be identical to the ViVA platform. The data sets will be linked via the code.

*Goal adjustment*. The Goal Adjustment Scale will be used to measure individuals ability to disengage from unachievable goals or to orient themselves towards new goals [29]. The 10 items will be be rated on a five-point Likert scale ranging from 1 = *not at all true* to 5 = *very true*. The two subscales, Goal Disengagement and Goal Reengagement, comprise four and six items, respectively.

*Satisfaction*. The German version of the Client Satisfaction Questionnaire (ZUF-8) [30] will be used to measure general satisfaction with the intervention. The eight items will be assessed on a four-point Likert scale ranging from 1 = *most unfavourable* to 4 = *most positive*. The working relationship between the consultant and the participant will be recorded with the German-language short version of the Working Alliance Inventory (WAI-SR) [31]. It will be completed by both the consultant and the participant at T2, T3, and T4. Satisfaction will be assessed only in the VA-V group.

*Rumination*. The German version of the rumination scale [32] of the Rumination-Reflection Questionnaire (RRQ [33]) will be used to obtain information about ruminative cognitive patterns, with 12 items on a 5-point scale measuring agreement.

*Baseline variables:*

*Sociodemographics.* Sociodemographic data (e.g. age, gender, and marital status) and basic medical data (e.g. time of diagnosis and tumour type/stage) will be collected according to the short form of the Psycho-Oncological Basic Documentation (PO-Bado-KF) [34].

*Diagnosis.* The Structured Clinical Interview for DSM-5, Clinician Version (SCID-5-CV) [35], a semistructured interview guide, will be used in a 2-h online diagnostic appointment that takes place in both study arms after the premeasurement but before inclusion in the study. Video-based interviews will be conducted by trained persons with at least a Bachelor’s degree in psychology, and the results will be documented in paper form. The diagnosing persons will be randomly assigned to the participants. The diagnosis made on the basis of the interview is then transferred to the ViVA platform by the diagnosing person. For quality assurance, diagnostic interviews will be videographed. A psychological psychotherapist will supervise the interviews.

#### Participant timeline

The study schedule, including enrolment, intervention, and data assessment, is shown in Fig. 1. Enrolled participants will receive an initial mental distress screening (NCCN Distress Thermometer). Individuals who report a distress score $\ge 5$ will complete further baseline questionnaires and a video-based diagnostic interview to complete the SCID. Individuals who report a distress score $< 5$ will be excluded from further participation. Once the presence of a mental disorder has been verified on the basis of this diagnostic interview, the participants will be randomly assigned to one of the two study arms. Individuals excluded according to the distress thermometer or SCID will receive information about possible contact points in the event of future cancer-related distress.

All participants will then have a first appointment with their randomly assigned consultant. If the participants are assigned to the intervention group, the first manualised intervention session will be conducted. If the participants are assigned to the control group, a supportive talk including feedback on the diagnosis and two further supportive talks will be provided.

Surveys using the above-mentioned standardised instruments will take place at five measurement points (before, during and directly after the intervention as well as 6 months after the intervention). Questionnaires for process evaluation (ZUF-8, WAI-SR) will be administered at the two interim measurements after the fourth and eighth sessions in the VA-V group. The measurements will take place online via the ViVA platform, and the participants will be reminded to complete the questionnaires. It is important for the project that the participants will respond as soon as possible (preferably within 1–3 days). The ViVA platform will send a reminder after 3 days and again after 6 days.

#### Sample size

Following the guidelines for psycho-oncological diagnostics [5], the Hospital Anxiety and Depression Scale (HADS) is defined as the primary endpoint for testing the effectiveness of the intervention. For the sample calculation, a mean effect of $d = 0.5$ of the VA-V versus the active control is assumed to be clinically relevant. This assumption is based on a review of the effects of psycho-oncological interventions compared to those of controls [36]. The sample size was calculated using SampSize (epiGenesys. A University of Sheffield company https://app.sampsize.org.uk). For the superiority hypotheses, an $\alpha$-error probability of .05, a power of $1- \beta = .80$, a mean effect of $d = .5$ (standardised mean difference), and a population $SD = 1$ (also standardised) are assumed. SampSize is used to calculate the sample size for a superiority trial with a parallel design and normally distributed outcome [37]. This results in $n = 51$ per group as a minimum requirement. Based on an estimated dropout rate of 30 % (e.g. [38]), $n = 73$ participants per group will be required. We aim to include $n = 146$ participants in the trial.

#### Recruitment

Participants are recruited at the Rehazentrum Oberharz Clausthal-Zellerfeld, Lower-Saxony, Germany, by university staff. The recruitment process will be supported by clinic staff (e.g. scheduling the information session on the patients’ calendar). Short presentations in a group setting will introduce patients to the study. The planned recruitment start date is 23 October 2023. The target sample size is $n = 146$. After reaching this sample size, recruitment is completed. If the target sample size is not reached, recruitment will be discontinued on 31 December 2024.

Potential participants will receive verbal information about the study project as well as written study information with a data protection declaration, information on video recordings, and two copies of the consent form (see Additional Files 2 and 3). The study information also explicitly states that people without relevant psychosocial distress cannot participate in the study and that participants in the German S3-Guideline group receive supportive talks but no intervention sessions of value-based behavioural activation. The signed consent form, including consent to videography of diagnostic and treatment appointments as well as the data protection declaration, will be a prerequisite for participation in the study. The participants will be informed that the videography will serve the purpose of quality assurance. Any further evaluation of the video material for research purposes will only take place if the participants expressly agree to this point separately in the declaration of consent. Consent forms will be signed by both the participant and the recruiting person, with one copy remaining with the participant. Any questions that may arise can be posed during the information event or afterwards via the contact details of the study staff provided on the information material. Participants will receive their personal access data to the ViVA platform via the contact details they have provided.

### Assignment of interventions

#### Sequence generation

Sequence generation will be achieved through covariate-adaptive randomisation according to gender (male, female, diverse) and type of tumour disease (4 types: breast, gastrointestinal, gynaecological and urogenital, other) by using an interactive web response system. All tumour types of diverse persons will be combined in one category due to the low expected sample size. Male breast cancers will be assigned to the category *other tumours* due to the low expected sample size. Information for this covariate adaptive randomisation will be collected at the baseline measurement through the PO-Bado-KF [34]. To achieve covariate adaptive randomisation, the first 36 individuals out of 143 (25 %) will be allocated completely at random. Starting with participant 37, allocation will be conducted by minimisation [39, 40].

#### Allocation concealment mechanism

To achieve allocation concealment, covariate-adaptive randomisation will be automated through an interactive web response system on the ViVA platform. Allocation will take place after the baseline assessment and diagnostic interview. The project staff cannot predict the randomisation result. The programming will be conducted by an external service provider (Consulting Partner Hannover GmbH).

#### Implementation

The allocation sequence will be generated as described above by an external service provider (Consulting Partner Hannover GmbH). Participants will be enrolled by project staff. The interactive web response system on the ViVA platform will assign participants to interventions.

#### Blinding

Participants cannot be blinded to their allocation since psychosocial interventions are identified naturally for both patients and counsellors. Baseline assessment will be conducted prior to allocation to minimise possible bias.

### Data collection, management, and analysis

#### Data collection methods

Invitations and reminders for the questionnaires will be sent by e-mail and/or SMS. In addition, the diagnostic interview and all sessions in the VA-V will be videographed. The videography of the diagnostic interview will be used for quality assurance. The research data and videographies used for this study will be stored for a period of 10 years in the Department of Clinical Psychology and Psychotherapy at the University of Hildesheim. The written consent forms will also be stored for 10 years. Contact data will be deleted after 36 months. Participants who discontinued the interventions will be asked to nevertheless respond to the remaining questionnaires. The reasons for drop-out will be assessed.

With the help of the ViVA platform, the data collection will be automated. The technical basis is the open-source software Limesurvey on the Linux Apache MySql server. In addition, fear of cancer recurrence will be recorded daily over 10 days with a separate platform on which the German translation of the FCR7 will be implemented. The platform will technically and in terms of data protection be identical to the ViVA platform. The data records will be linked via the participant code.

#### Data management

The data management plan was published on the German Clinical Trial Register website of this trial and will be updated every 6 months. Each participant consent form will be marked with a participant code. The consent form with contact details will thus allow for an assignment of personal data and code. Further data (from the ViVA platform, FCR platform, SCID interview, and videography) will be marked with this participant code and stored separately from the personal data. Thus, all the data intended for scientific evaluation will be stored exclusively in pseudonymous form. The data will, therefore, be de facto anonymous after deletion of the personal data at the end of the trial. Participants will have the right to request the deletion of personal data necessary for scientific evaluation without giving reasons, before the study is completed.

After the deletion of the personal data at the end of the study, deletion will no longer be possible, as the data will be de facto anonymous. Publication of the raw data is not intended. For ethical reasons, the raw data will only be available upon request. Interested researchers will be able to apply to the corresponding author of the study publication for access to the data. All requests will be reviewed by the data protection officer of the University of Hildesheim.

#### Statistical methods

The statistical evaluations for the analysis of the superiority of the VA-V will be carried out as an intention-to-treat analysis via multilevel structural equation models (measurement points nested in persons). Missing values will be estimated using the maximum likelihood method (expectation-maximisation algorithm). The stratification variables (diagnosis and gender) will be considered predictors. To check whether the intervention is implemented as planned, a process evaluation will be conducted, and fidelity will be recorded. In this context, treatment satisfaction and the assessment of the therapeutic relationship, as well as various parameters, such as dropout rates, will be considered. In addition, the predictive significance of participant characteristics (e.g. goal adjustment, treatment satisfaction) for therapy success (HADS) or discontinuation will be determined via regression analyses.

### Monitoring

#### Data monitoring

The data will be monitored by MR. Additionally, regular team meetings (every 2 weeks) and supervision sessions (every 1–2 weeks) will be conducted. Adverse events, including data findings as well as occurrences in the consultations, will be reported to the supervising psychological psychotherapist (CH) and the primary sponsor and principal investigator of the study (CK). Interim analyses will only apply to the outcomes that the consultants need for the intervention sessions. No stopping rules have been defined for this trial.

#### Harms

The study will not give rise to any additional health risks, impairments, or burdens that go beyond what would be expected in everyday life or in the context of another survey or psycho-oncological counselling. It is not assumed that participation in the study will have negative effects on participants, such as anxiety, exhaustion, or discomfort, which go beyond what is experienced in other psycho-oncological consultations. Nevertheless, all adverse events will be documented. Should negative effects occur, these will be closely monitored by a psychological psychotherapist. If a study participant’s symptoms worsen and require more intensive psychotherapeutic treatment, psychological psychotherapists will be available at the university outpatient clinic.

## Discussion

This study will evaluate whether the VA-V is effective at reducing distress compared to the German S3-Guideline-compliant aftercare. The consequences of a tumour disease can lead to a relevant and possibly chronic impairment of abilities and of occupational and social participation. A successful adaptation of personal values and goals to a changed life situation can mitigate these effects, as it enables those affected to optimise their everyday life based on their existing abilities and to maintain or restore their ability to work.

One strength of this study is its robust design and follow-up assessment at 6 months allowing evaluation of the long-term effects of VA-V. However, there will also be several challenges in this study. In particular, enrolling a sufficiently large sample may pose a difficulty, which we hope to overcome by recruiting individuals in person at the Rehazentrum Oberharz. To ensure treatment integrity, extensive training prior to implementation, regular supervision, and documentation of the implementation will be essential strategies. These measures will be supported by videographies. If the intervention is discontinued, an attempt will be made to still include the participant in the data collection. Selective dropout in the control group may pose a problem. Nevertheless, three supportive talks constitute more comprehensive aftercare than CAU and may, therefore, motivate participants to complete their study participation.

In summary, this will be the first study to test a manualised VA-V in the aftercare of persons with tumour disease in a randomised controlled trial. If the VA-V proves to be effective, the results will be relevant for improving the aftercare of persons with tumour disease and, as such, be of high public health relevance. The manual of the intervention will be published after a positive evaluation of its effectiveness. Assuming the effectiveness of psycho-oncological treatment, dissemination will be guaranteed by the following points: The manualised intervention will be disseminated through further training offers, including DKG-certified curricula. Various professional groups interested in psycho-oncology, with and without a licence to practise, will be addressed. The knowledge gained regarding the effectiveness and acceptance of video-based treatment will hold great potential for improving psychotherapeutic care in rural areas and for special patient groups with limited mobility.

## Trial status

This is protocol version 1.0 of 7 November 2023. The first participant consented to study participation on 30 October 2023. Recruitment will be completed by 31 December 2024 (or earlier if targeted participant numbers are reached). Important protocol modifications will be communicated to the Ethics Committee and the German Clinical Trials Register, and a protocol amendment will be submitted to TRIALS. The results of the trial will be published in an open access journal once the data analyses will be complete (ca. end of 2025). Participants who wish to receive the results, will be informed of the trial results in an information letter sent via e-mail.

### Supplementary information

**Additional file 1.** SPIRIT Checklist.

**Additional file 2.** Informed consent materials (original version).

**Additional file 3.** Informed consent materials (English version).

## Data Availability

Data are available upon request due to ethical restrictions. Interested researchers may submit requests to the corresponding author for access to the data. All requests will be assessed by the Data Protection Officer of the University.
