# Supplementary material for: Outpatient follow-up of tumour diseases through video-based value-oriented behavioural activation (ViVA): study protocol for a randomised controlled trial
Source: Trials. 2024 Feb 14;25:121. doi: 10.1186/s13063-024-07953-w (PMC10865710; doi:10.1186/s13063-024-07953-w)
Supplement: Supplementary file 2 — Additional file 2. Informed consent materials (original version). [file 13063_2024_7953_MOESM2_ESM.pdf]

„Ambulante Nachsorge bei Tumorerkrankungen durch videobasierte werteorientierte Verhaltensaktivierung – eine randomisiert kontrollierte Studie / ViVA“

Studieninformation

Sehr geehrte Damen und Herren,

mit dieser Studieninformation möchten wir Ihnen einen Überblick über die Studie **„Ambulante Nachsorge bei Tumorerkrankungen durch videobasierte werteorientierte Verhaltensaktivierung – eine randomisiert kontrollierte Studie / ViVA“** geben. Die Studie wird von der Arbeitsgruppe Klinische Psychologie und Psychotherapie der Universität Hildesheim und dem „Rehazentrum Oberharz“ als Kooperationspartner durchgeführt.

Wir möchten Sie einladen, an dieser klinischen Studie teilzunehmen. Bevor Sie sich entscheiden teilzunehmen, ist es wichtig, zu verstehen, warum diese Studie durchgeführt wird und was sie beinhaltet. Bitte nehmen Sie sich Zeit, um die folgenden Informationen sorgfältig zu lesen. Fragen Sie uns, wenn Ihnen etwas unklar ist, oder wenn Sie weitere Informationen wünschen. Nehmen Sie sich ggf. Zeit zu entscheiden, ob Sie teilnehmen möchten. Die Teilnahme ist freiwillig.

Wenn Sie teilnehmen möchten, unterschreiben Sie bitte die beigefügte Einverständniserklärung. Geben Sie die unterschriebene Einverständniserklärung entweder direkt der Person, von der Sie diese Unterlagen erhalten haben, der Kontaktperson an der Rehaklinik Oberharz oder nutzen Sie einen frankierten Rückumschlag an die Universität Hildesheim. Sie können Ihr Einverständnis jederzeit und ohne Begründung oder Nachteile für Sie wieder zurückziehen.

1. Was sind Ziel und Zweck dieser Studie?

Das Forschungsprojekt zielt darauf ab, die Wirksamkeit der videobasierten werteorientierten Verhaltensaktivierung (VA-V) bei Tumorerkrankten mit psychischer Belastung im Vergleich zur Nachbehandlung entsprechend der Leitlinie zur psychoonkologischen Versorgung zu untersuchen. Die Leitlinie ist eine systematisch entwickelte Handlungsempfehlung, die Ärzt:innen und Patient:innen bei der Entscheidungsfindung über die angemessene Behandlung einer Krankheit unterstützt. Das Projekt soll zur verbesserten psychoonkologischen Versorgung von Tumorerkrankten beitragen.

Sie werden zufällig entweder in die VA-V-Gruppe oder in eine Leitliniengruppe gelost.
In der Leitliniengruppe erhalten Sie eine ausführliche klinische Diagnostik in Form eines Interviews, das zur Erhebung von relevanten Informationen im Zusammenhang mit Ihrer Erkrankung eingesetzt wird. Eine Rückmeldung dazu erhalten Sie im Rahmen eines Gesprächs. Zusätzlich erhalten Sie zwei weitere unterstützende Gespräche.

Werden Sie zufällig in die VA-V-Gruppe gelost, erhalten Sie zusätzlich zur Diagnostik zwölf Interventionssitzungen in Form von videobasierten Gesprächen, die auf Ihre persönlichen Werte und Bedürfnisse zugeschnitten sind.

Was passiert in der videobasierten werteorientierten Verhaltensaktivierung (VA-V)?

Während der Projektlaufzeit wird das aus der Depressionsbehandlung entliehene Konzept der werteorientierten Verhaltensaktivierung in der ambulanten Tumornachsorge erprobt. Teilnehmer:innen erhalten dabei in zwölf manualisierten Nachsorgesitzungen eine systematische Unterstützung bei der Anpassung individueller Bedürfnisse und Ziele. Manualisiert bedeutet, dass die Einzelschritte der Therapie in einem Handbuch genau beschrieben wurden. Durch die Nutzung der Videosprechstunde können Teilnehmer:innen und Behandler:innen direkt miteinander kommunizieren, ohne dass ein Kontakt in Präsenz erforderlich ist. Die Sitzungen der VA-V-Gruppe finden üblicherweise wöchentlich statt.

Was passiert in der leitlinienkonformen Nachbehandlung?

In der Nachbehandlung nach der Leitlinie zur psychoonkologischen Versorgung (S3) erhalten Sie eine ausführliche Rückmeldung der Diagnostik. Darüber hinaus bekommen Sie in den zwei weiteren unterstützenden Gesprächen Anregungen zum Umgang mit Problemen, die mit der Krebserkrankung einhergehen können, sowie bei Bedarf Unterstützung in weiteren, individuell festzulegenden Bereichen (z.B. Umgang mit Fatigue, Entspannungsverfahren).

2. Was bringt mir die Teilnahme an der Studie?

Durch die Teilnahme an der Studie erhalten Sie in jedem Fall eine umfangreiche Diagnostik, auch wenn wir in der betreffenden Sitzung keine relevante psychische Belastung bei Ihnen feststellen. Als Teilnehmer:in der VA-V-Gruppe erhalten Sie zwölf Sitzungen. In diesen Sitzungen werden Sie dabei unterstützt, die Veränderungen in Ihrem Leben, die durch Ihre Erkrankung und deren Behandlung entstanden sind, zu bewältigen und Ihr psychisches Wohlbefinden zu verbessern.
In der leitlinienkonformen Nachbehandlung erhalten Sie drei unterstützende Gespräche.

3. Wie läuft die Studie ab?

Bei einer Teilnahme an der Studie kommen auf Sie die folgenden Schritte zu:
1) Sie erhalten eine **Studieninformation mit Datenschutzerklärung und Einverständniserklärung**. Wenn Sie teilnehmen möchten, füllen Sie die Einverständniserklärung aus.
2) Sie erhalten Ihre **persönlichen Zugangsdaten** für das Studienportal (ViVA-Plattform) und schließen dort die **Registrierung** für die Studie ab. Die Zugangsdaten erhalten Sie per E-Mail oder als SMS (je nach Wunsch). Die digitale ViVa-Plattform ermöglicht eine für die Teilnehmer:innen maßgeschneiderte und auf dem Bildschirm des Computers sichtbare Benutzeroberfläche, die bei der Durchführung der einzelnen Studienschritte unterstützt.
3) Sie bekommen ebenfalls per E-Mail oder SMS einen Link für den **Fragebogen zum Studienbeginn**. Diesen füllen Sie auf der ViVA-Plattform online aus.
4) Wir führen mit Ihnen ein sogenanntes **Klinisches Interview** (ausführliche Diagnostik Ihrer aktuellen psychischen Situation) in einer Videosprechstunde.

5) Sofern eine akute psychische Belastung besteht, werden Sie **zufällig** einer der beiden **Gruppen** (VA-V-Gruppe oder Leitliniengruppe) zugeteilt. Sofern keine aktuell relevante Belastung besteht, endet die Studienteilnahme nach diesem Schritt.
6) Sofern Sie weiter an dieser Studie teilnehmen, erhalten Sie zwölf bzw. drei Sitzungen, zwischendurch werden Sie zweimal gebeten, online auf der ViVA-Plattform einen Fragebogen auszufüllen.
7) Nach der letzten Sitzung folgt der **Fragebogen zum Ende der Sitzungen**; diesen füllen Sie wieder online auf der ViVA-Plattform aus.
8) Weitere sechs Monate später erfolgt noch einmal eine **Nachbefragung**, dazu erhalten Sie wieder einen persönlichen Link und füllen einen Fragebogen auf der ViVA-Plattform aus.

4. Kann ich an der Studie teilnehmen?

Wir suchen tumorerkrankte Personen im Alter zwischen 18 und 75 Jahren, die eine Rehabilitationsmaßnahme im Rehazentrum Oberharz durchlaufen und eine hohe subjektive Belastung empfinden, die dann anhand eines diagnostischen Interviews bestätigt wird. Sie sollten über ausreichend gute Deutschkenntnisse verfügen. Ausschlusskriterien sind das Vorliegen einer bipolaren Störung, Schizophrenie oder anderer wahnhafter Störungen sowie einer Substanz- oder Essstörung. Zu den Ausschlusskriterien zählt auch eine laufende ambulante Psychotherapie oder die kurzfristige Aussicht auf einen Psychotherapieplatz (nicht: Wartelistenplatz).
Für die videobasierten Behandlungstermine benötigen Sie folgende technische Voraussetzungen:

- ein Endgerät wie Computer, Laptop oder Tablet mit Bildschirm,
- eine Webcam (ggf. im Endgerät integriert), Mikrofon und Lautsprecher,
- einen modernen Browser (**Chrome, Edge Chromium, Firefox oder Safari** werden unterstützt) und
- eine ausreichend stabile Internetverbindung.

Das Gespräch läuft über RED-Connect als Videodienstanbieter. Dieser Anbieter erfüllt besondere Sicherheitsstandards, damit alles, was zwischen Behandler:in und Teilnehmer:in besprochen wird, vor Dritten geschützt bleibt.

5. Warum werden Videoaufzeichnungen gemacht?

Wir möchten im Rahmen der ViVA-Studie Videoaufzeichnungen anfertigen und erklären Ihnen ausführlich auf Seite 9 in den „Information zu Videoaufnahmen“ wofür wir die Aufnahmen nutzen möchten.

6. Gibt es Risiken dieser Studie?

Bei dieser Studie gibt es keine gesundheitlichen Risiken, Beeinträchtigungen oder Belastungen, die über das im Alltag zu erwartende Maß während einer psychoonkologischen Beratung hinausgehen. Bei dieser Datenerhebung werden keine invasiven Methoden (d.h. bspw. keine Blutabnahme, keine Verabreichung radioaktiver Mittel, etc.) angewandt.
Sollten Sie während der Studie Nebenwirkungen bemerken, bitten wir Sie, diese der:dem Behandler:in mitzuteilen.

7. Ist die Studienteilnahme freiwillig?

Die Teilnahme an dieser Studie ist freiwillig. Sie können diese Studie jederzeit und ohne Angaben von Gründen abbrechen. Darüber hinaus hat dies weder eine nachteilige Auswirkung auf Ihre Weiterbehandlung der Tumorerkrankung noch anderweitige Konsequenzen. Ihre medizinische Behandlung wird sowohl hinsichtlich der Anzahl als auch hinsichtlich der Art und des Inhalts fortgesetzt, unabhängig davon, ob Sie an der Studie teilnehmen oder nicht.

8. Ist die Studie versichert?

Bei dieser Studie fällt keine Zusatzversicherung an, da keine zusätzlichen, belastenden oder direkt auf den Körper einwirkenden Maßnahmen oder Eingriffe auf Sie zukommen.

9. Wie ist der Datenschutz geregelt?

Alle erhobenen Daten werden entsprechend den geltenden Datenschutzbestimmungen vor dem Zugriff Unbefugter geschützt. Ausführliche Informationen finden Sie in der Datenschutzerklärung und den Datenschutzbestimmungen für Videoaufzeichnungen. Die Studie wurde durch die Ethikkommission der Universität Hildesheim genehmigt.

10. Kontakt und Fragen

**Studienleitung** an der Universität Hildesheim:
{Contact Details}
**Kontaktdaten für Anfragen** an der Universität Hildesheim
{Contact Details}
**Kooperationspartnerinnen** im Rehazentrum Oberharz**:**
{Contact Details}

„Ambulante Nachsorge bei Tumorerkrankungen durch videobasierte werteorientierte Verhaltensaktivierung – eine randomisiert kontrollierte Studie / ViVA"

Datenschutzerklärung

Was geschieht mit Ihren Daten?

Bei dieser Studie werden Befragungsdaten (Online-Fragebogen, klinisches Interview) und Kontaktdaten erhoben, verarbeitet und auf elektronischen Datenträgern gespeichert. Die Weitergabe, Speicherung und Auswertung dieser Daten erfolgen nach gesetzlichen Bestimmungen. Die im Rahmen der Studie erhobenen Daten unterliegen der Schweigepflicht und den datenschutzgesetzlichen Bestimmungen.

Einverständniserklärungen:

Die Einverständniserklärungen werden an die Universität Hildesheim geschickt. Hier werden Sie für 10 Jahre nach Studienende aufbewahrt und anschließend datenschutzkonform vernichtet.

Kontaktdaten (Name, E-Mail-Adresse, Mobilnummer und Adresse) mit Teilnahme-Code:

Mit der Online-Registrierung zur Studie willigen Sie in die Datenverarbeitung Ihrer personenbezogenen Daten ein. Hierzu wird durch die Projektmitarbeiter:innen ein zufällig generierter Teilnahme-Code vergeben. Ihre Kontaktdaten (E-Mail-Adresse, Mobilfunknummer und Adresse) werden getrennt von Ihrem Antwortdatensatz gespeichert. Über Ihren Teilnahme-Code sind beide Datensätze verknüpfbar. Bei Abschluss der Studie werden Ihre Kontaktdaten und Ihr verbundener Teilnahme-Code gelöscht. Ihre Studiendaten sind nach diesem Schritt defacto anonymisiert.

Befragungsdaten (Online-Fragebogen, klinisches Interview):

Die Fragebögen werden digital und pseudonym erfasst. Das heißt, dass in dem Fragebogen nicht nach Ihren Kontaktdaten gefragt wird und diese nur über den Teilnahme-Code mit dem Fragebogen in Verbindung gebracht werden können.
„Pseudonymisieren" bedeutet gemäß Art. 4 Nr. 5 DSGVO *„die Verarbeitung personenbezogener Daten in einer Weise, dass die personenbezogenen Daten ohne Hinzuziehung zusätzlicher Informationen nicht mehr einer spezifischen betroffenen Person zugeordnet werden können, sofern diese zusätzlichen Informationen gesondert aufbewahrt werden und technischen und organisatorischen Maßnahmen unterliegen, die gewährleisten, dass die personenbezogenen Daten nicht einer identifizierten oder identifizierbaren natürlichen Person zugewiesen werden."*

Ihren Antworten werden automatisiert Zeitstempel hinzugefügt, d.h. wir können sehen wann Sie eine Frage beantwortet haben und wann Sie den Fragebogen begonnen und beendet haben.
Die Auswertung wird nach wissenschaftlichen Gesichtspunkten erfolgen. Eine Auswertung auf der Ebene von Einzelpersonen wird nicht durchgeführt. Der pseudonymisierte und nach Ende der Studie faktisch anonyme Datensatz wird verschlüsselt gespeichert. In dieser Form wird er für 10 Jahren nach der Veröffentlichung gesichert und anschließend gelöscht.

Wer hat Zugang zu den Daten?

Nur die am Projekt beteiligten Personen am Institut für Psychologie der Universität Hildesheim, Arbeitsgruppe Klinische Psychologie und Psychotherapie sowie der zuständigen IT-Firma Consulting Partner Hannover GmbH, haben nach datenschutzrechtlicher Unterweisung und Unterzeichnung einer Schweigepflichtserklärung Zugang zu den Kontakt- und Befragungsdaten.

Wie werden die Daten geschützt?

Der Zugang zu den Kontaktdaten und Befragungsdaten ist passwortgeschützt (Rechner und Datei) und nur auf die am Projekt beteiligten Personen am Institut für Psychologie der Universität Hildesheim, Arbeitsgruppe Klinische Psychologie und Psychotherapie beschränkt. Die Consulting Partner Hannover GmbH hat als IT-Verwalter der ViVA-Plattform Zugriffsmöglichkeiten auf die Plattform, um bei technischen Problemen zu helfen. Als weisungsabhängiger Dienstleister würde ein Zugriff auf die Daten nur nach Anweisung durch die Projektbeteiligten der Arbeitsgruppe Klinische Psychologie und Psychotherapie der Universität Hildesheim erfolgen; dies ist im Projekt nicht vorgesehen. Die digitale Speicherung und Verarbeitung daraus generierter Datensätze erfolgt anonymisiert und ist ebenfalls passwortgeschützt. Zentral werden die Datensätze auf geschützten Servern gespeichert (NAS).
Die Einverständniserklärungen - und sofern notwendig Datenausdrucke in Papierform - werden in einem Studienordner in einem abgeschlossenen Schrank in einem abgeschlossenen Studienbüro der Arbeitsgruppen Klinische Psychologie und Psychotherapie der Universität Hildesheim aufbewahrt. Zugriff haben auch hier nur Personen der Arbeitsgruppen Klinische Psychologie und Psychotherapie der Universität Hildesheim beschränkt.

In welcher Form werden die Ergebnisse veröffentlicht?

Die Auswertung und Veröffentlichung der Ergebnisse der Befragung wird in anonymisierter Form erfolgen, d.h. ohne dass Daten einzelnen Personen zugeordnet werden können. Wir nehmen keine Auswertung von Einzelfällen vor.
Nach Abschluss der Studie könnten die Daten zur wissenschaftlichen Nachnutzung an andere wissenschaftliche Mitarbeiter:innen weitergegeben werden. Dies erfolgt ausschließlich in anonymisierter Form und unter den datenschutzrechtlichen Bedingungen, die hier dargelegt werden.

Datenschutzrechte im Überblick:

Widerspruchs- und Beseitigungsmöglichkeiten, Auskunftsrecht, Recht auf Widerruf der datenschutzrechtlichen Einwilligungserklärung und Recht auf Löschung:

Sowohl die Kontaktdaten als auch die Befragungsdaten können bis zum Ende der Studie gelöscht werden, wenn die datenschutzrechtliche Einwilligungserklärung widerrufen wird. Wir können einer Einzelperson Auskunft darüber geben, ob ihre Daten gespeichert sind.
Nach dem Ende der Studie ist eine Löschung nicht mehr möglich, da die Befragungsdaten selbst anonymisiert verarbeitet werden und die Kontaktdaten gelöscht wurden. Die Antworten können nicht mehr bestimmten Personen zugeordnet werden. Entsprechend können wir, wenn die datenschutzrechtliche Einwilligungserklärung dann widerrufen wird, keine Daten mehr löschen und einer Einzelperson auch keine Auskunft mehr darüber geben, ob ihre Daten gespeichert sind.

Recht auf Beschwerde bei einer Aufsichtsbehörde:

Unbeschadet eines anderweitigen verwaltungsrechtlichen oder gerichtlichen Rechtsbehelfs steht Ihnen das Recht auf Beschwerde bei einer Aufsichtsbehörde, insbesondere in dem Mitgliedstaat Ihres Aufenthaltsorts, Ihres Arbeitsplatzes oder des Orts des mutmaßlichen Verstoßes, zu, wenn Sie der Ansicht sind, dass die Verarbeitung der Sie betreffenden personenbezogenen Daten gegen die Datenschutz-Grundverordnung (DSGVO) verstößt.

Verantwortlicher Datenschutzbeauftragter:
apl. Prof. Dr. Thomas Mandl, Universität Hildesheim, Universitätsplatz 1,
31141 Hildesheim, E-Mail: {Contact Details}

Verantwortliche der Datenverarbeitung:
Univ.-Prof. Dr. phil. Christoph Kröger, Universitätsplatz 1, 31141 Hildesheim, E-Mail: {Contact Details}

Bei Datenschutzverstößen haben Sie bei den folgenden Datenschutz-Aufsichtsbehörden ein Beschwerderecht:

Landesbeauftragte für den Datenschutz Niedersachsen (poststelle@lfd.niedersachsen.de) oder Bundesdatenschutzbeauftragter: Die Bundesbeauftragte für den Datenschutz und die Informationsfreiheit (poststelle@bfdi.bund.de, Tel: +49 (0) 228-997799-0).

„Ambulante Nachsorge bei Tumorerkrankungen durch videobasierte werteorientierte Verhaltensaktivierung – eine randomisiert kontrollierte Studie / ViVA"

Information zu Videoaufnahmen

Wir möchten im Rahmen der ViVA-Studie Videoaufzeichnungen anfertigen und erklären Ihnen im Folgenden, wofür wir die Aufnahmen nutzen möchten.

Die Videoaufzeichnungen der Diagnostik- und Behandlungssitzungen dienen der Qualitätssicherung: Sie helfen uns dabei, die Umsetzung des Behandlungshandbuchs unter Leitung einer Psychologischen Psychotherapeutin zu überprüfen (Behandlungsintegrität und Supervision). Somit dienen sie insbesondere angesichts des Umgangs mit sensiblen Themen zu Ihrem Schutz und sind daher verpflichtend für die Teilnahme.

Darüber hinaus würden wir gerne eine Auswertung einzelner Inhalte (zum Beispiel eines Gedankenexperiments) in anonymisierter Form zu wissenschaftlichen Zwecken durchführen. Dafür ist jedoch auf der beiliegenden Einverständniserklärung Ihre gesonderte Zustimmung erforderlich. Diese Zustimmung ist freiwillig und Sie können unabhängig davon an der Studie teilnehmen.

Der Zugang zu den Videoaufzeichnungen ist passwortgeschützt (Rechner und Datei) und nur auf die am Projekt beteiligten Personen am Institut für Psychologie der Universität Hildesheim, Arbeitsgruppe Klinische Psychologie und Psychotherapie beschränkt. Die Consulting Partner Hannover GmbH hat als IT-Verwalter der ViVA-Plattform Zugriffsmöglichkeiten auf die Plattform um bei technischen Problemen zu helfen. Als weisungsabhängiger Dienstleister würde ein Zugriff auf die Videoaufzeichnungen nur nach Anweisung durch die Projektbeteiligten der Arbeitsgruppe Klinische Psychologie und Psychotherapie der Universität Hildesheim erfolgen; dies ist im Projekt nicht vorgesehen. Diese Personen unterliegen der gesetzlichen Schweigepflicht gemäß §203 StGB und den geltenden Datenschutzvorschriften.

Gemäß Leitlinie 17 „Archivierung" der DFG-Leitlinien zur Sicherung guter wissenschaftlicher Praxis werden die für diese Studie verwendeten Videographien für einen Zeitraum von zehn Jahren in der Arbeitsgruppe Klinische Psychologie der Universität Hildesheim archiviert, um eine nachhaltige Aufbewahrung der Daten zu gewährleisten.

Sollten Sie mit Videoaufnahmen für die genannten Zwecke einverstanden sein, bitten wir Sie, dies auf der nachfolgenden Einwilligungserklärung anzugeben und zu unterzeichnen. Das Einverständnis kann jederzeit ohne Angaben von Gründen bei Ihrem:Ihrer Behandler:in oder der Studienleitung widerrufen werden. Die Videoaufnahmen werden dann gelöscht. Es entstehen Ihnen bei einer Ablehnung der Teilnahme oder wenn Sie Ihr Einverständnis zur forschungsbezogenen Auswertung widerrufen keine Nachteile oder negativen Auswirkungen bezüglich Ihres weiteren Versorgungsstandards außerhalb der Studie.

„Ambulante Nachsorge bei Tumorerkrankungen durch videobasierte werteorientierte Verhaltensaktivierung – eine randomisiert kontrollierte Studie / ViVA"

Einverständniserklärung zur Teilnahme

Ich bin ausführlich und verständlich über das Wesen, die Bedeutung und die Tragweite der Studie „Ambulante Nachsorge bei Tumorerkrankungen durch videobasierte werteorientierte Verhaltensaktivierung – eine randomisiert kontrollierte Studie / ViVA" von

____

(Name der/des aufklärenden Mitarbeiter:in in Druckbuchstaben) aufgeklärt worden.

Mir wurde die Möglichkeit gegeben, Fragen zu stellen, und diese wurden zufriedenstellend beantwortet. Ich habe darüber hinaus den Text der Studieninformation, der Datenschutzerklärung sowie der Information zu Videoaufnahmen gelesen und verstanden. Ich hatte ausreichend Zeit, mich für die Teilnahme zu entscheiden.

Mir ist bewusst, dass die Teilnahme an dieser Studie freiwillig erfolgt. Mir ist außerdem bekannt, dass ich meine Einwilligung jederzeit ohne Angabe von Gründen widerrufen kann (mündlich oder schriftlich), ohne dass mir daraus Nachteile für meine weitere medizinische/psychotherapeutische Behandlung entstehen.

DATENSCHUTZ UND NUTZUNG IHRER DATEN

Mir ist bekannt, dass bei wissenschaftlichen Studien persönliche Daten und medizinische Befunde erhoben werden. Die Speicherung und Auswertung dieser studienbezogenen Daten erfolgen nach gesetzlichen Bestimmungen und setzt vor der Teilnahme an dieser Studie folgende freiwillig abgegebene Einwilligungserklärung voraus. Das heißt, dass ich ohne die nachfolgende Einwilligung nicht an der Studie teilnehmen kann.

1. Ich willige ein, dass im Rahmen dieser Studie meine Kontaktdaten und personenbezogenen Daten, insbesondere Angaben über meine Gesundheit, über mich erhoben werden und in Papierform sowie auf elektronischen Datenträgern bei der auf Seite 1 genannten Arbeitsgruppe Klinische Psychologie und Psychotherapie der Universität Hildesheim aufgezeichnet werden. Mir ist bekannt, dass die von mir erhobenen Daten pseudonymisiert werden. Eine Auswertung und Veröffentlichung erfolgt nur mit anonymisierten Daten. Zudem werden alle Sitzungen in der VA-V und das diagnostische Interview zur Qualitätssicherung videographiert. Die Auswertung der Videographien zu Forschungszwecken erfolgt nur, wenn ich untenstehend gesondert zustimme.

2. Ich bin darüber aufgeklärt worden, dass ich mich bei Fragen an den in der Datenschutzerklärung genannten Datenschutzbeauftragten wenden kann und ein Beschwerderecht bei der in der Datenschutzerklärung genannten Datenschutz-Aufsichtsbehörden habe.

3. Ich bin darüber aufgeklärt worden, dass ich jederzeit die Teilnahme an der Studie beenden kann. Nach dem Widerruf meiner Einwilligungserklärung werden keine weiteren Daten von mir im Rahmen dieser Studie erhoben. Alle bis zum Zeitpunkt meines Widerrufs gespeicherten personenbezogenen Daten inklusive der Videoaufnahmen werden zu den unten genannten regulären Terminen gelöscht. Ich habe das Recht, die unmittelbare Löschung aller bereits erhobenen Daten zu verlangen.
Nach Löschung der Kontaktdaten (nach 36 Monaten) ist eine Löschung der pseudonymisierten Daten nicht mehr möglich, da eine Zuordnung von Person und Pseudonym nur bis zu diesem Zeitpunkt möglich ist.

4. Ich bin darauf hingewiesen worden, dass ich das Recht habe, Auskunft (einschließlich unentgeltlicher Überlassung einer Kopie) über meine personenbezogenen Daten zu erhalten sowie ggf. deren Berichtigung oder Löschung zu verlangen.

5. Ich willige ein, dass meine faktisch anonymen Daten nach Beendigung oder Abbruch der Studie mindestens 10 Jahre aufbewahrt werden. Danach werden diese Daten gelöscht, soweit nicht gesetzliche, satzungsmäßige oder vertragliche Aufbewahrungsfristen entgegenstehen. Die Kontaktdaten werden bereits nach 36 Monaten gelöscht.

Wenn Sie teilnehmen möchten, müssen Sie diesen Punkt ankreuzen.

☐ Ich erkläre mich mit der Teilnahme an der Studie einverstanden. Dies umfasst auch die Aufzeichnung der Diagnostik- und Behandlungssitzungen auf Video zur Qualitätssicherung und zur Supervision. Ich habe die Studieninformation, die Datenschutzerklärung, die Information zu Videoaufnahmen und ein Exemplar der unterschriebenen Einverständniserklärung erhalten. Das Original verbleibt in der **Arbeitsgruppe Klinische Psychologie und Psychotherapie** der Universität Hildesheim.

Diesem Punkt können Sie zustimmen oder nicht zustimmen. Sie können ihn auch offenlassen und zu einem späteren Zeitpunkt mit den Behandler:innen klären.

☐ Ich bin damit einverstanden, dass meine Video-Aufzeichnungen im Rahmen forschungsbezogener Auswertungen anonymisiert genutzt werden und somit die Schutzbelange meiner Person gewürdigt bleiben. Es erhalten nur Mitarbeiter:innen der Abteilung Klinische Psychologie und Psychotherapie Einblick, die mit der jeweiligen Forschungsfrage befasst sind und außerdem der gesetzlichen Schweigepflicht unterliegen.

☐ Meine Videoaufzeichnungen dürfen **nicht** zu forschungsbezogenen Auswertungen genutzt werden.

Durch die/den Teilnehmer:in persönlich in Druckbuchstaben auszufüllen:

Bitte teilen Sie uns Ihren Vor- und Nachnamen mit

Bitte teilen Sie uns Ihre Adresse (Straße, Hausnummer)

(Postleitzahl und Wohnort) mit

Bitte teilen Sie uns für die Kontaktaufnahme Ihre E-Mail-Adresse **und**

Ihre Mobilfunknummer (wenn keine Mobilfunknummer, dann Festnetznummer) mit

Bitte teilen Sie uns für die Zusendung des Befragungslinks den Weg der bevorzugten Kontakt-aufnahme mit:

☐ E-Mail

☐ SMS

______________________________ ____

Ort, Datum Unterschrift Teilnehmer:in

Durch die/den aufklärende:n Mitarbeiter:in persönlich auszufüllen:

Ich habe das Aufklärungsgespräch geführt und die Einwilligung des/der Teilnehmer:in erhalten.

______________________________ ____

Ort, Datum Unterschrift Mitarbeiter:in
