# Supplementary material for: Outpatient follow-up of tumour diseases through video-based value-oriented behavioural activation (ViVA): study protocol for a randomised controlled trial
Source: Trials. 2024 Feb 14;25:121. doi: 10.1186/s13063-024-07953-w (PMC10865710; doi:10.1186/s13063-024-07953-w)
Supplement: Supplementary file 3 — Additional file 3. Informed consent materials (English version). [file 13063_2024_7953_MOESM3_ESM.pdf]

<u>English translation of text of the original document</u>

"Outpatient follow-up of tumour diseases through video-based value- oriented behavioural activation - a randomised controlled trial / ViVA"
Information on the study

Dear Ladies and Gentlemen,

with this study information we would like to give you an overview of the study "***Outpatient follow-up of tumour diseases through video-based value-oriented behavioural activation - a randomised controlled trial / ViVA*** ". The study is being conducted by the Clinical Psychology and Psychotherapy Working Group of the University of Hildesheim and the "Rehazentrum Oberharz" as cooperation partner.

We would like to invite you to participate in this clinical trial. Before you decide to take part, it is important to understand why this study is being done and what it involves. Please take time to read the following information carefully. Ask us, if anything is unclear to you or if you would like to have more information. If necessary, take time to decide whether you would like to take participate. Participation is voluntary.

If you would like to participate, please sign the enclosed consent form. Either give the signed consent form directly to the person from whom you received these documents, to the contact person at the Rehazentrum Oberharz or use a stamped return envelope to the University of Hildesheim. You can withdraw your consent at any time and without justification or disadvantages for you.

1. What are the aim and purpose of this study?

The research project aims to investigate the effectiveness of video-based value-oriented behavioural activation (VA-V) for tumour patients with psychological distress in comparison to follow-up treatment according to the guideline on psycho-oncological care. The Guideline is a systematically developed recommendation for action that supports doctors and patients in making decisions about the appropriate treatment of a disease. The project is intended to contribute to improved psycho-oncological care for tumour patients.

You will be randomly assigned to either the VA-V group or a guideline group.
In the guideline-group, you will receive a detailed clinical diagnosis on the basis of an interview that will be used to collect relevant information related to your condition. You will receive feedback on this during a supportive talk. In addition, you will receive two further supportive talks.

If you are randomly assigned to the VA-V group, you will receive twelve intervention sessions in the form of video-based consultations tailored to your personal values and needs in addition to the diagnostic interview.

What happens in video-based value-oriented behavioural activation (VA-V)?

During the project, the concept of value-oriented behavioural activation borrowed from depression treatment will be tested in outpatient tumour aftercare. Participants receive systematic support in adapting individual needs and goals in twelve manualised aftercare sessions. Manualised means that the individual steps of the therapy were described in detail in a manual. Through the use of video consultation, participants and consultants can communicate directly with each other without the need for face-to-face contact. The sessions of the VA-V group usually take place weekly.

What happens in guideline-compliant follow-up treatment?

In the follow-up treatment according to the guideline on psycho-oncological care (S3), you will receive detailed feedback on the diagnosis. In addition, in the two further supportive talks you will receive suggestions on how to deal with problems that may be associated with the cancer and, if necessary, support in other areas that will be determined individually (e.g. dealing with fatigue, relaxation procedures).

2. What do I get out of participating in the study?

By participating in the study, you will in any case receive a comprehensive diagnostic assessment, even if we do not diagnose any relevant psychological distress during the diagnostic interview. As a participant in the VA-V group, you will receive twelve sessions. In these sessions, you will be supported in coping with the changes in your life that have occurred as a result of your illness and its treatment, and in improving your psychological well-being.

In the guideline-compliant follow-up treatment, you will receive three supportive talks.

3. How does the study work?

If you participate in the study, the following steps will apply to you:

1) You will receive a study information leaflet with privacy policy and consent form. If you would like to participate, you can fill out the consent form.

2) You will receive your personal access data for the study portal (ViVA platform) and complete the registration for the study there. You will receive the access data by e-mail or as an SMS (depending on your preference). The digital ViVa platform enables a user interface that is customised for the participants and visible on the computer screen, which supports them in carrying out the individual study steps.

3) You will also receive a link to the first study questionnaire by email or SMS. You fill this out online on the ViVA platform.

4) We will conduct a so-called clinical interview (detailed diagnostics of your current mental situation) with you in a video consultation.

5) If there is acute psychological distress, you will be randomly assigned to one of the two groups (VA-V group or guideline group). If there is no current relevant distress, participation in the study ends after this step.

6) If you continue to participate in this study, you will receive twelve or three sessions, respectively, and in between you will be asked twice to complete a questionnaire online on the ViVA platform.

7) After the last session, you will be asked to complete the end-of-sessions questionnaire, again online on the ViVA platform.

8) Another six months later there will be another follow-up survey, again you will receive a personal link and fill in a questionnaire on the ViVA platform.

4. Can I participate in the study?

We are looking for people with tumours aged between 18 and 75 who are undergoing a rehabilitation measure at the Oberharz Rehabilitation Centre and experience a high level of subjective distress, which is then confirmed on the basis of a diagnostic interview. They should have a sufficiently good knowledge of German. Exclusion criteria are the presence of bipolar disorder, schizophrenia or other delusional disorders as well as a substance use or eating disorder. Exclusion criteria also include ongoing outpatient psychotherapy or the short-term prospect of a psychotherapy place (not: place on the waiting list).
For the video-based treatment appointments you need the following technical requirements:

- a terminal device such as a computer, laptop or tablet with a screen,
- a webcam (integrated in the end device, if applicable), microphone and loudspeaker,
- a modern browser (Chrome, Edge Chromium, Firefox or Safari are supported) and
- a sufficiently stable internet connection.

The appointments run via RED-Connect as the video service provider. This provider meets special security standards so that everything that is discussed between the consultant and the participant remains protected from third parties.

5. Why are video recordings made?

We would like to make video recordings as part of the ViVA study and explain in detail on page 9 in the "Information on video recordings" what we would like to use the recordings for.

6. Are there any risks associated with this study?

In this study, there are no health risks, impairments or burdens that go beyond what can be expected in everyday life during psycho-oncological treatment. No invasive methods (e.g. no blood sampling, no administration of radioactive substances, etc.) are used in this data collection. If you notice any side effects during the study, please report them to your consultant.

7. Is participation in the study voluntary?

Participation in this study is voluntary. You can discontinue this study at any time and without giving any reason. Furthermore, this will have neither an adverse effect on your further treatment of the tumour disease nor any other consequences. Your medical treatment will

continue, both in terms of number and type and content, regardless of whether you participate in the study or not.

8. Is the study insured?

There is no additional insurance for this study, as there are no additional, stressful measures or interventions that directly affect your body.

9. How is data protection regulated?

All data collected is protected from access by unauthorised persons in accordance with the applicable data protection regulations. Detailed information can be found in the privacy policy and the data protection regulations for video recordings. The study was approved by the Ethics Committee of the University of Hildesheim.

10. Contact and questions

**Study head** at the Universität Hildesheim:
{Contact Details}
**Contact details for enquiries** at the Universität Hildesheim
{Contact Details}
**Cooperation partner** at the Rehazentrum Oberharz**:**
{Contact Details}

"Outpatient follow-up of tumour diseases through video-based value-oriented behavioural activation - A randomised controlled Trial / ViVA"
Data Protection Declaration

What happens with your data?

In this study, survey data (online questionnaire, clinical interview) and contact data are collected, processed and stored on electronic data carriers. The transfer, storage and evaluation of this data is carried out in accordance with legal regulations. The data collected within the scope of the study are subject to confidentiality and the provisions of data protection law.

Consent forms:

The consent forms will be sent to the University of Hildesheim. They are stored there for 10 years after the end of the study and then destroyed in accordance with data protection regulations.

Contact details (name, e-mail address, mobile number and address) with participation code:

By registering online for the study, you consent to the data processing of your personal data. For this purpose, a randomly generated participation code will be assigned by the project staff. Your contact details (e-mail address, mobile phone number and address) will be stored separately from your response data. Both data sets can be linked via your participation code. At the end of the study, your contact details and your linked participation code will be deleted. Your study data are de facto anonymised after this step.

Survey data (online questionnaire, clinical interview):

The questionnaires are collected digitally and pseudonymously. This means that the questionnaire does not ask for your contact details and these can only be linked to the questionnaire via the participation code.
"Pseudonymisation" means, according to Art. 4 No. 5 DSGVO, *"the processing of personal data in such a way that the personal data can no longer be attributed to a specific data subject without the addition of further information, provided that such additional information is kept separately and is subject to technical and organisational measures which ensure that the personal data are not attributed to an identified or identifiable natural person."*

Time stamps are automatically added to your answers, i.e. we can see when you answered a question and when you started and finished the questionnaire.
The evaluation will be carried out according to scientific criteria. An evaluation at the level of individuals will not be carried out. The pseudonymised and, after the end of the study, de facto anonymous data set will be stored in an encrypted form. It will be saved in this form for 10 years after publication and then deleted.

Who has access to the data?

Only the persons involved in the project at the Institute of Psychology at the University of Hildesheim, Working Group Clinical Psychology and Psychotherapy and the responsible IT company Consulting Partner Hannover GmbH, have access to the contact and survey data after being instructed in data protection law and signing a confidentiality agreement.

How is the data protected?

Access to the contact data and survey data is password-protected (computer and file) and restricted only to the people involved in the project at the Institute of Psychology at the University of Hildesheim, Clinical Psychology and Psychotherapy Working Group. Consulting Partner Hannover GmbH, as IT administrator of the ViVA platform, has access to the platform to help with technical problems. As a service provider dependent on instructions, access to the data would only take place following instructions from those involved in the project in the Clinical Psychology and Psychotherapy Working Group at the University of Hildesheim; this is not envisaged in the project. The digital storage and processing of data sets generated from this is anonymised and is also password-protected. The data sets are stored centrally on protected servers (NAS).
The consent forms - and, if necessary, data printouts in paper form - are stored in a study folder in a locked cabinet in a locked study office of the Clinical Psychology and Psychotherapy Working Group at the University of Hildesheim. Here too, access is restricted to persons from the Clinical Psychology and Psychotherapy Working Group at the University of Hildesheim.

In what form will the results be published?

The evaluation and publication of the results of the survey will take place in anonymised form, i.e., without data being able to be assigned to individual persons. We do not evaluate individual cases.
After completion of the study, the data could be passed on to other academic staff members for subsequent academic use. This will only be done in anonymous form and under the data protection conditions set out here.

Data protection rights at a glance:

Possibilities of objection and removal, right to information, right to revoke the declaration of consent under data protection law and right to deletion:

Both contact data and survey data can be deleted until the end of the study if the declaration of consent under data protection law is revoked. We can provide an individual with information on whether their data is stored.
After the end of the study, deletion is no longer possible because the survey data itself is processed anonymously and the contact data have been deleted. The answers can no longer be assigned to specific individuals. Accordingly, if the declaration of consent under data protection law is revoked then, we can no longer delete any data or provide an individual with information about whether their data is stored.

Right to complain to a supervisory authority:

Without prejudice to any other administrative or judicial remedy, you have the right to lodge a complaint with a supervisory authority, in particular in the Member State of your residence, place of work or the place of the alleged infringement, if you consider that the processing of personal data concerning you infringes the *Datenschutz-Grundverordnung* (DSGVO).

Responsible Data Protection Officer:
apl. Prof. Dr. Thomas Mandl, Universität Hildesheim, Universitätsplatz 1,
31141 Hildesheim, e-mail: {Contact Details}

Responsible for data processing:
Univ.-Prof. Dr. phil. Christoph Kröger, Universitätsplatz 1, 31141 Hildesheim, e-mail:
{Contact Details}

In the event of data protection violations, you have the right to complain to the following data protection supervisory authorities:

Landesbeauftragte für den Datenschutz Niedersachsen (poststelle@lfd.niedersachsen.de)
or Bundesbeauftragte für den Datenschutz und die
Informationsfreiheit (poststelle@bfdi.bund.de, Tel: +49 (0) 228-997799-0).

"Outpatient follow-up of tumour diseases through video-based value-oriented behavioural activation - A randomised controlled trial / ViVA"

Information on Video Recordings

We would like to take video recordings as part of the ViVA study and explain below what we would like to use the recordings for.

The video recordings of the diagnostic and treatment sessions serve quality assurance purposes: they help us to check the implementation of the treatment manual under the supervision of a psychological psychotherapist (treatment integrity and supervision). Thus, they serve to protect you, especially in view of the handling of sensitive topics, and are therefore obligatory for participation.

In addition, we would like to carry out an evaluation of individual contents (for example, a thought experiment) in anonymised form for scientific purposes. However, your separate consent is required for this on the enclosed consent form. This consent is voluntary and you can participate in the study regardless of it.

Access to the video recordings is password-protected (computer and file) and restricted only to the persons involved in the project at the Institute of Psychology at the University of Hildesheim, Clinical Psychology and Psychotherapy Working Group. Consulting Partner Hannover GmbH, as the IT administrator of the ViVA platform, has access to the platform to help with technical problems. As a service provider dependent on instructions, access to the video recordings would only take place on the instructions of those involved in the project from the Clinical Psychology and Psychotherapy Working Group at the University of Hildesheim; this is not intended in the project. These persons are subject to the legal duty of confidentiality according to §203 StGB and the applicable data protection regulations.

In accordance with Guideline 17 "Archiving" of the DFG Guidelines for Safeguarding Good Scientific Practice, the videographies used for this study will be archived for a period of ten years in the Clinical Psychology Working Group at the University of Hildesheim in order to ensure sustainable storage of the data.

If you agree to video recordings for the above-mentioned purposes, please indicate this on the following consent form and sign it. The consent can be revoked at any time without giving reasons by contacting your consultant or the study management. The video recordings will then be deleted. You will not suffer any disadvantages or negative consequences with regard to your further standard of care outside the study if you refuse to participate or if you withdraw your consent to the research-related evaluation.

"Outpatient follow-up of tumour diseases through video-based value-oriented behavioural activation - A randomised controlled trial / ViVA"
Declaration of consent to participation

I have been informed in detail and in a comprehensible manner about the nature, significance and scope of the study "Outpatient follow-up of tumour diseases through video-based value-oriented behavioural activation - A randomised controlled trial / ViVA" by

____

(name of the informing staff member in block capitals).

I was given the opportunity to ask questions and these were answered satisfactorily. I have also read and understood the text of the study information, the data protection declaration and the information on video recordings. I have had sufficient time to decide whether to participate.

I am aware that participation in this study is voluntary. I am also aware that I can withdraw my consent at any time without giving reasons (verbally or in writing) without any disadvantages for my further medical/psychotherapeutic treatment.

DATA PROTECTION AND USE OF YOUR DATA
I am aware that personal data and medical findings are collected during scientific studies. The storage and evaluation of this study-related data is carried out according to legal regulations and requires the following voluntarily given declaration of consent before participation in this study. This means that I cannot participate in the study without the following consent.

1. I consent to my contact details and personal data, in particular information about my health, being collected about me in the course of this study and recorded in paper form and on electronic data carriers at the Clinical Psychology and Psychotherapy Working Group of the University of Hildesheim named on page 1. I am aware that the data collected from me will be pseudonymised. An evaluation and publication will only take place with anonymised data. In addition, all sessions in the VA-V and the diagnostic interview are videographed for quality assurance purposes. The evaluation of the videographs for research purposes will only take place if I give my separate consent below.
2. I have been informed that I can contact the data protection officer named in the data protection declaration if I have any questions and that I have a right of appeal to the data protection supervisory authorities named in the data protection declaration.
3. I have been informed that I can terminate my participation in the study at any time. After revoking my declaration of consent, no further data will be collected from me as part of this study. All personal data stored up to the time of my revocation, including video recordings, will be deleted on the regular dates mentioned below. I have the right to demand the immediate deletion of all data already collected. After deletion of the contact data (after 36 months), deletion of the pseudonymised data is no longer

possible, as an assignment of person and pseudonym is only possible until this point in time.

4. I have been informed that I have the right to receive information (including the free provision of a copy) about my personal data and, if necessary, to demand its correction or deletion.
5. I agree that my de facto anonymous data will be kept for at least 10 years after the end or termination of the study. After this period, the data will be deleted unless there are legal, statutory or contractual retention periods to the contrary. Contact data will already be deleted after 36 months.

If you would like to participate, you must tick this item.

○ I agree to participate in the study. This also includes the recording of the diagnostic and treatment sessions on video for quality assurance and supervision. I have received the study information, the data protection declaration, the information on video recordings and a copy of the signed consent form. The original document remains in the Clinical Psychology and Psychotherapy Working Group at the University of Hildesheim.

You can agree or disagree with this aspect. You can also leave it open and clarify it with the consultant at a later time.

○ I agree that my video recordings will be used anonymously in the context of research-related evaluations and that my personal privacy will thus be protected. Only staff members of the Clinical Psychology and Psychotherapy Working Group who are involved in the respective research question and who are also subject to the legal duty of confidentiality will be given access.

○ My video recordings may not be used for research-related evaluations.

To be filled out in print letters by the participant: Please provide us with your first and last name

Please provide us with your address (street, house number)

(Postal code and city)

Please provide us with your email address for contacting you **and**

your mobile number (if no mobile number, then landline number)

Please let us know your preferred method of contact for sending the survey link:

□ E-Mail

□ SMS

____

Place, date Signature participant

To be filled out personally by the informing staff member:
I have conducted the briefing and obtained the consent of the participant.

______________________________ ____

Place, date Staff member's signature
